# Pentacyclic Triterpenes from Olive Leaves Formulated in Microemulsion: Characterization and Role in De Novo Lipogenesis in HepG2 Cells

**DOI:** 10.3390/ijms241512113

**Published:** 2023-07-28

**Authors:** Marzia Vasarri, Donatella Degl’Innocenti, Laura Albonetti, Anna Rita Bilia, Maria Camilla Bergonzi

**Affiliations:** 1 Department of Experimental and Clinical Biomedical Sciences, University of Florence, Viale Morgagni 50, 50134 Florence, Italy; marzia.vasarri@unifi.it (M.V.); donatella.deglinnocenti@unifi.it (D.D.); 2 Department of Chemistry, University of Florence, Via U. Schiff 6, 50519 Sesto Fiorentino, Italy; laura.albonetti@stud.unifi.it (L.A.); ar.bilia@unifi.it (A.R.B.)

**Keywords:** pentacyclic triterpenes, *Olea europaea* L., microemulsion, PAMPA, HepG2 cells, intracellular lipid accumulation

## Abstract

*Olea europaea* L. leaves contain a wide variety of pentacyclic triterpenes (TTPs). TTPs exhibit many pharmacological activities, including antihyperlipidemic effects. Metabolic alterations, such as dyslipidemia, are an established risk factor for hepatocellular carcinoma (HCC). Therefore, the use of TTPs in the adjunctive treatment of HCC has been proposed as a possible method for the management of HCC. However, TTPs are characterized by poor water solubility, permeability, and bioavailability. In this work, a microemulsion (ME) loading a TTP-enriched extract (EXT) was developed, to overcome these limits and obtain a formulation for oral administration. The extract-loaded microemulsion (ME-EXT) was fully characterized, assessing its chemical and physical parameters and release characteristics, and the stability was evaluated for two months of storage at 4 °C and 25 °C. PAMPA (parallel artificial membrane permeability assay) was used to evaluate the influence of the formulation on the intestinal passive permeability of the TTPs across an artificial membrane. Furthermore, human hepatocarcinoma (HepG2) cells were used as a cellular model to evaluate the effect of EXT and ME-EXT on de novo lipogenesis induced by elevated glucose levels. The effect was evaluated by detecting fatty acid synthase expression levels and intracellular lipid accumulation. ME-EXT resulted as homogeneous dispersed-phase droplets, with significantly increased EXT aqueous solubility. Physical and chemical analyses showed the high stability of the formulation over 2 months. The formulation realized a prolonged release of TTPs, and permeation studies demonstrated that the formulation improved their passive permeability. Furthermore, the EXT reduced the lipid accumulation in HepG2 cells by inhibiting de novo lipogenesis, and the ME-EXT formulation enhanced the inhibitory activity of EXT on intracellular lipid accumulation.

## 1. Introduction

**Figure 1:**
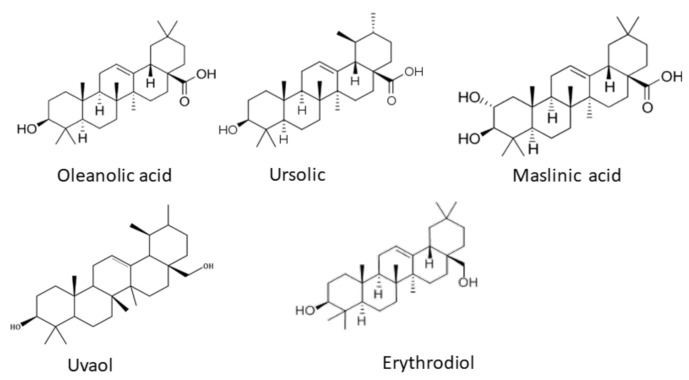
TTPs chemical structure.

Among the secondary metabolites of plants, pentacyclic triterpenes (TTPs) are crucial for plant defense, and they have positive impacts on human health. The fruits and the leaves of *Olea europaea* L. contain various sterols and triterpenoids [1,2,3]. Numerous health benefits have been attributed to the presence of TTPs in olive leaves [4,5,6]. For this reason, olive leaf bioactive compounds have attracted growing attention [2]. The TTPs content is higher in the leaves than in the fruit and is dependent on the variety. The leaf contains high amounts of oleanolic acid (3.0–3.5% dry weight); a significant concentration of maslinic acid (0.50–0.75% dry weight); and minor levels of ursolic acid (0.20–0.25% dry weight), erythrodiol, and uvaol, which are present in comparable amounts, in a range of 0.05 and 0.15% dry weight [7] (Figure 1). There is a growing demand for TTPs in the market because they have the potential to be incorporated into new functional foods, cosmetics, and drugs. They have many pharmacological activities; however, their poor water solubility, permeability, and bioavailability are limiting factors for their therapeutic application [8,9,10,11,12,13]. Various effects of TTPs have been extensively investigated in the literature: anti-inflammatory, antioxidant, anti-hyperglycemic, anti-hyperlipidemic, cardio- and hepatoprotective, chemoprotective effects, as well as effects on the epidermal cells and SARS-CoV-2 [14,15,16,17,18,19,20,21,22,23,24,25,26,27,28]. Numerous studies have been published on the anticancer activity of TTPs in various cancer models, including liver cancer, skin cancer, colon cancer, lung cancer, breast cancer, pancreatic cancer, and leukemia [14]. For instance, oleanolic acid (OA) prevents hepatocellular carcinoma progression in transplanted HepG2 cells and in human hepatoma HepG2 cells [29,30,31].

Globally, hepatocellular carcinoma (HCC) is the second leading cause of cancer death faced by public health organizations around the world [32]. Metabolic alterations are an established risk factor for HCC. Although the risk factors for the development of HCC in non-alcoholic fatty liver disease (NAFLD) patients are not entirely clear, additional metabolic conditions such as diabetes mellitus, obesity, dyslipidemia, and increased blood glucose levels have been related to the development of NAFLD into liver cancer [33,34]. Lipids enhance tumor activity, regulating a variety of biological functions [35]. De novo lipogenesis is a hallmark of cancer, is often enhanced in solid tumors, and reduces the reliance of cancer cell growth on exogenous fatty acids [36].

HCC is characterized by an upregulation of fatty acid synthesis-related genes, including fatty acid synthase (FASN), which catalyzes the de novo synthesis of long-chain fatty acids [37]. The inhibition of FASN has been reported to have antitumor effects [38]. The development and progression of liver cancer have been associated with lipid dysregulation [35].

Although some treatments for HCC are available, improvements are needed to reduce the mortality rate and increase the quality of life of patients. Indeed, despite these therapeutic alternatives, the recurrence rate at 2 years can reach 50% [39]. Adjuvant therapies may thereby affect the long-term survival of these patients by preventing HCC recurrence after (or before) successful curative therapeutic procedures. Due to their pharmacological activities, the use of TTPs in the adjunctive treatment of HCC was proposed as a possible opportunity for the management of HCC.

The use of herbal extracts has increased worldwide, due to their nutritional and therapeutic properties. Despite outstanding results in experimental in vitro models, herbal extracts often show a lower or negligible in vivo activity, due to their poor solubility, resulting in poor absorption and hence poor bioavailability. This could be due to partial or total destruction in the highly acidic pH of the stomach. To date, novel drug delivery systems have made it possible to improve the bioavailability of plant extracts, by improving solubility, protecting against toxicity, sustaining delivery and protection from physical and chemical degradation, enhancing pharmacological activity and intracellular uptake, and modifying the biodistribution. In addition, plant extracts present difficulties, because of the wide variety of compounds they contain. Oral absorption of hydrophobic extracts can be significantly improved using lipid-based non-particulate drug delivery systems, and microemulsion systems appear the most promising. This study aimed to develop an oral formulation that can overcome this limited solubility and oral permeability, by increasing the therapeutic efficacy of a TTP-enriched extract (EXT) obtained from olive leaves [40,41]. This extract is certainly more advantageous for commercial applications than the single OA, which requires a lengthy purification processes. An extract-loaded microemulsion (ME-EXT) was prepared and fully characterized, and its stability was evaluated during 2 months of storage at 4 °C and 25 °C. Furthermore, a PAMPA assay (parallel artificial membrane permeability assay) was performed, to check the ability of the formulation to improve the intestinal passive permeability of the extract’s constituents across an artificial membrane that mimics that of the intestine [42]. Elevated blood glucose levels associated with metabolic disorders can be risk factors for tumor progression. Therefore, here, HepG2 human hepatoma cells were exposed to 25 mM D-Glucose (high glucose conditions, HG), to study HG-related lipid accumulation, as reported in the literature [43]. Cells exposed to physiological glucose (normal glucose conditions, NG, 5 mM) were used as the experimental control condition. Experimental assays were performed on HepG2 cells, to assess the effect of EXT and ME-EXT on de novo lipogenesis induced by elevated glucose levels. For this purpose, FASN expression levels and intracellular lipid accumulation were detected.

## 2. Results and Discussion

### 2.1. Preparation and Characterization of ME-EXT

**Figure 2:**
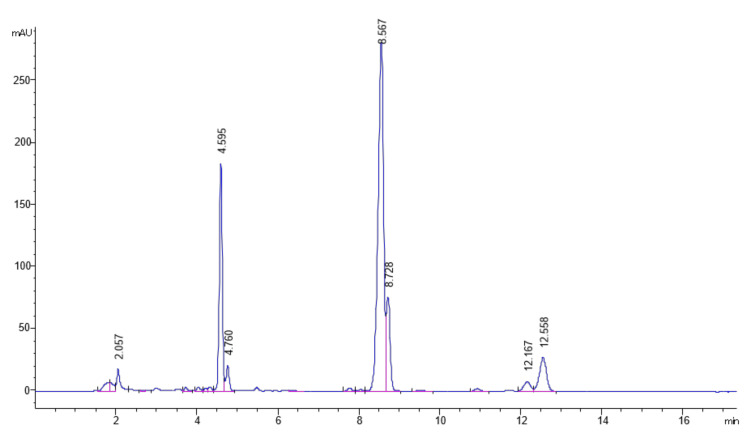
Chromatographic profile (210 nm) of the EXT. Maslinic acid: 4.59 min, oleanolic acid: 8.56 min, ursolic acid: 8.72 min, uvaol: 12.16 min, erythrodiol: 12.55 min.

The analysis with high-performance liquid chromatography coupled with a photodiode-array detector (HPLC-DAD) of EXT allowed identifying the following TTPs: maslinic acid, oleanolic acid, ursolic acid, uvaol and erythrodiol (Figure 2); the percentage of TTPs was 63.04 ± 1.06% *w*/*w*.

A phytocomplex, that is a whole or partially purified extract of a plant constituent, offers greater efficacy and advantages over a single isolated ingredient, thanks to synergistic interactions [44,45]. Among the various drug delivery systems, ME is considered the optimal choice for the oral delivery of extracts. ME is able to transform a dried extract into an oral dosage form, to overcome the limited oral bioavailability, by improving its solubility, stability, intestinal permeability, and oral bioavailability. The oil-in-water microemulsions were formulated with food-acceptable components, to increase the solubility and stability, and improve the intestinal permeability of the extract constituents [40,41,46]. The excipients were selected from food-grade ingredients, generally recognized as safe, using liquid lipids and tensides, which greatly increased the solubility of the OA and phenol-rich extract, as previously reported [41,47,48,49,50]. Additionally, several excipients used, including Cremophor, Tween 80, and Transcutol, are P-gp inhibitors [51] and can modulate intestinal permeability. The field of existence of the ME was defined using a pseudo-ternary phase diagram. Transcutol HP and D-α-Tocopherol polyethylene glycol succinate (TPGS) were mixed at different ratios to obtain Smix. Then, different combinations of oily phase and Smix were considered and the mixtures were titrated with water, to define a transparent system. The ME domain (purple, Figure S1 of the Supplementary Materials) was determined through visual inspection; the light blue region was the emulsion region. The ME final composition was 67.5% Transcutol HP, 7.5% TPGS, 8% Capryol 90, and 17% water. The aqueous solubility of EXT at 25 °C was 9.14 ± 0.44 µg/mL; instead, ME-EXT solubilized 2 mg/mL (0.2% *w*/*w*) of EXT, significantly increasing its solubility.

Dynamic light scattering (DLS) and electrical light scattering (ELS) analyses of ME-EXT evidenced a homogenous system, with high ζ-potential. Empty ME showed a size of 115.50 ± 2.04 nm, PdI of 0.19 ± 0.00, and ζ-potential of −14.24 ± 0.41 mV (values are reported as mean ± SD of three independent experiments). The presence of EXT did not modify the physical parameters of the system, the dimensions were 107.82 ± 1.54 nm, and only a small increase in PdI value (0.24 ± 0.02) and a decrease in ζ-potential (−20.52 ± 0.41 mV) were found.

TEM analysis confirmed the DLS results (Figure S2 of the Supplementary Materials), showing non-aggregated droplets with a spherical shape.

### 2.2. In Vitro Release Study

**Figure 3:**
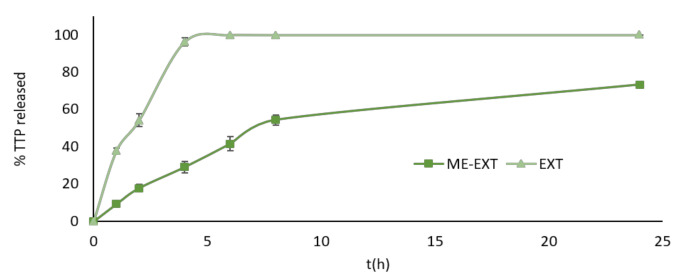
In vitro release profiles of TTPs from the solution and ME-EXT in EtOH:PBS (30:70 *v*/*v*). Values are reported as mean ± SD of three experiments.

A solution of EtOH:PBS (30:70 *v*/*v*) was used as release medium to perform the in vitro release experiments. ME demonstrated a significant effect on delaying the release of TTPs. The release from ME was slow and gradual compared to the release obtained with the solution (Figure 3). In the latter case, in fact, 96.24 ± 2.16% of TTPs had been released after 4 h, reaching 100% in the following hours. The release of the TTPs from ME-EXT was more gradual, with a 73.25 ± 1.76% released after 24 h.

The release of TTPs was also evaluated in simulated gastric fluid (SGF) and simulated intestinal fluid (SIF) (Figure S3 of the Supplementary Materials). The amount of TTPs released from the formulation was 20.79 ± 0.89% after 2 h of the experiment in SGF and 35.31 ± 1.68% after 6 h in SIF. The release pattern was slow and gradual in both fluids, similarly to the EtOH:PBS results. The ME formulation enhanced the EXT retention time, achieving a gradual release and protecting the EXT in the acid environment; the formulation released a smaller quantity of TTPs compounds compared to the amount released in the other two media, i.e., EtOH:PBS and SIF [47].

### 2.3. Stability Study over Time

**Figure 4:**
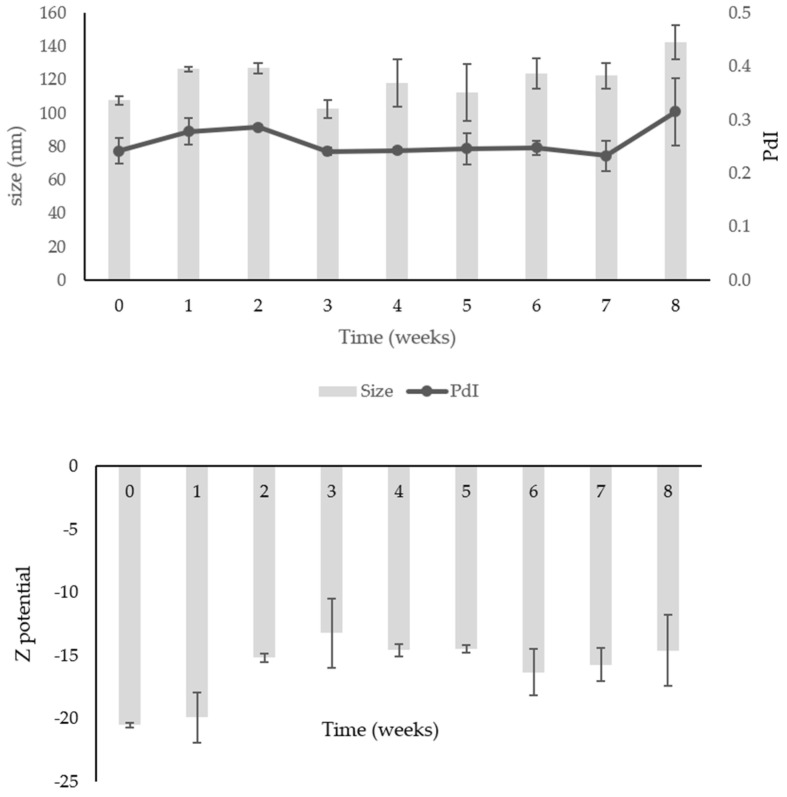
Physical stability (size, PdI, and ζ-potential) of ME-EXT at 25 °C. Data were obtained from the mean ± SD of three experiments.

**Figure 5:**
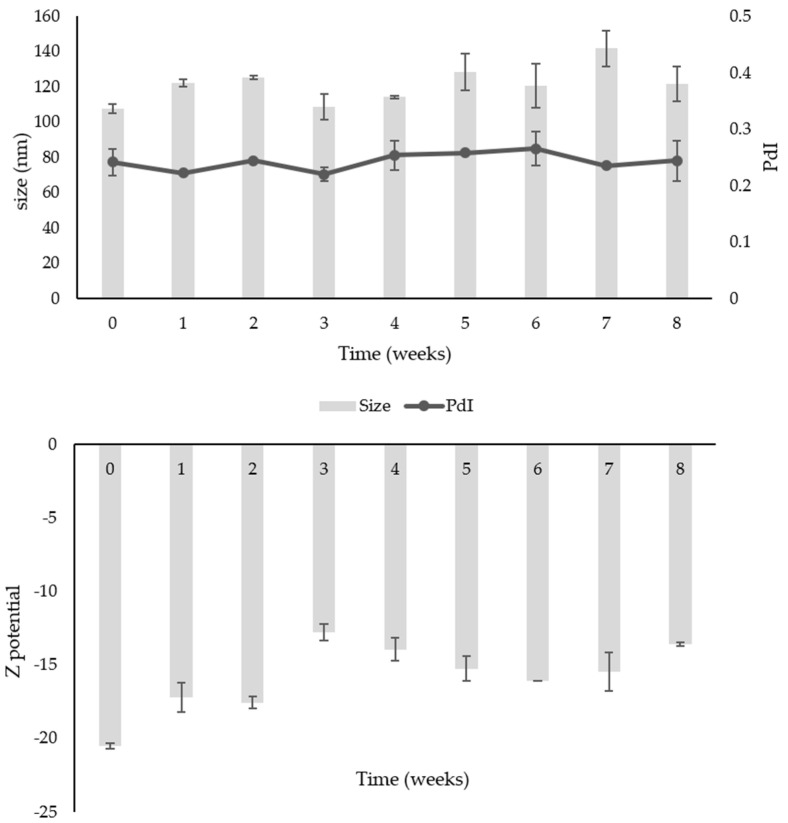
Physical stability (size, PdI, and ζ-potential) of ME-EXT at 4 °C. Data were obtained from the mean ± SD of three experiments.

The formulation was stable for 8 weeks at room temperature and +4 °C (Figure 4 and Figure 5), maintaining its physical characteristics and a high recovery. Regarding the storage at room temperature (Figure 4), a variation in size was observed in the first 7 weeks, from 102.61 ± 5.36 nm to 127.1 ± 3.11 nm, with an increase up to 142.65 ± 10.25 nm at week 8. The same trend was observed for the PdI, which remained constant for 7 weeks and slightly increased to 0.31 ± 0.06 after 8 weeks, always maintaining good physical characteristics. The ζ-potential remained around—15 mV throughout the test.

In the case of ME-EXT stored in the fridge, a slight increase in size during the test was observed, but this was less marked than at 25 °C. The dimensions ranged from 107.8 ± 2.55 nm to 121.65 ± 10.11 nm. The PdI had an average value of 0.24 ± 0.01, and the ζ-potential remained around—15 mV. These findings demonstrate the physical stability of the formulation for two months, proving that the storage at 4 ± 2 °C was the best condition to preserve the ME (Figure 5). Furthermore, the chemical stability was high, with a TTP recovery percentage after two months of 96.20% at 25 °C and 97.96% at 4 °C.

### 2.4. Parallel Artificial Membrane Permeability Assay (PAMPA)

PAMPA allows fast in vitro determination of the passive permeability of compounds through an artificial membrane that mimics the intestinal barrier. TTPs are not permeable molecules, and no permeation was observed after 6 h of test. The ME improved the passive permeation of EXT, with an effective permeability (Pe) value for TTPs of 1.20 × 10^−7^ ± 3.76 × 10^−8^ cm/s after 2 h and 1.02 × 10^−6^ ± 1.48 × 10^−7^ after 6 h. The recovery percentage was 94.26%. The rising Pe values highlighted an increase in passive permeation of TTPs, due to the components of the formulation acting as penetration enhancers across the simulated artificial intestinal epithelium, in addition to the fact that they enhanced the EXT solubility [40,47,50].

### 2.5. Effect of EXT and ME-EXT on HepG2 Cell Viability under HG Conditions

**Figure 6:**
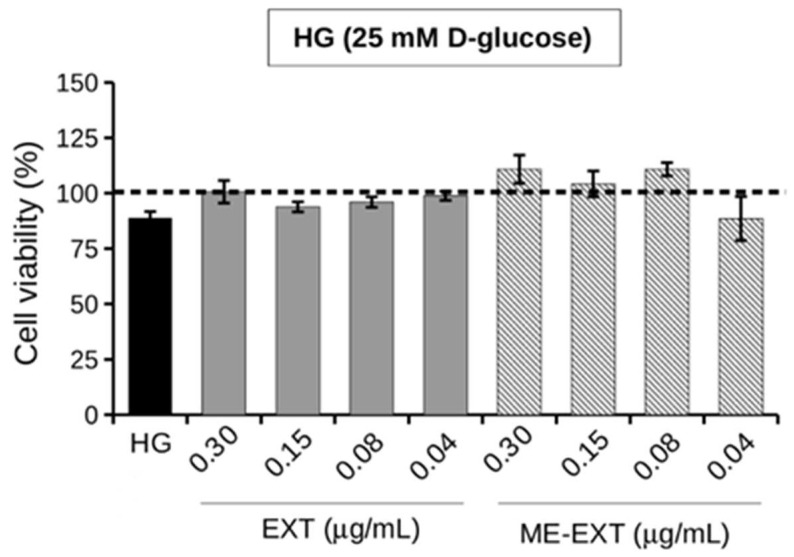
Cell viability of HepG2 cells treated with EXT or ME-EXT in the range of 0.30–0.04 µg/mL (*w*/*v*) EXT, under high-glucose conditions (HG, 25 mM D-glucose) for 24 h. Untreated cells exposed to HG were used as control. The values are given as percentages compared to the same cell treatments carried out under normal glucose conditions (NG, 5 mM D-glucose) represented on the graph by the dotted line. The data were obtained from the mean ± standard deviation of three experiments.

The MTT assay was used to determine the viability of HepG2 human hepatoma cells treated with EXT or ME-EXT in the range of concentrations of the extract between 0.3 and 0.04 µg/mL under high glucose conditions (HG, 25 mM D-Glucose, Figure 6). Cells exposed to normal glucose conditions (NG, 5 mM D-Glucose) in the absence or presence of EXT or ME-EXT were used as controls. Although high blood glucose levels are often associated with cellular damage and toxicity [52], cancer cells take advantage of glucose utilization to meet to their huge energy demand and for rapid proliferation and expansion. In this work it was observed that 24-h exposure to HG had no significant effect on HepG2 cell viability, which was comparable to NG-exposed cells, in agreement with previous work [43]. The presence of free EXT or ME-EXT also did not significantly influence cell viability, which was comparable to that of cells treated with free or delivered EXT under NG conditions. Vehicle ME caused no change in cell viability under HG conditions (Figure S4 of the Supplementary Materials). Given the lack of toxicity of free and ME-EXT during the 24-h exposure to HG, subsequent experiments were performed using EXT or ME-EXT at the two highest doses tested (0.30 and 0.15 µg/mL).

### 2.6. Effect of EXT and ME-EXT on Intracellular Lipid Accumulation in HepG2 Cells under HG Conditions

Lipid metabolism enables cancer cells to obtain energy, membrane components, and signaling molecules for proliferation, survival, invasion, metastasis, and in response to the impacts from the tumor microenvironment [53]. Increased lipid synthesis is a remarkable feature of cancer metabolism [54]. The literature describes an increased intracellular lipid accumulation in HepG2 cells under HG conditions within 24 h [43,55].

**Figure 7:**
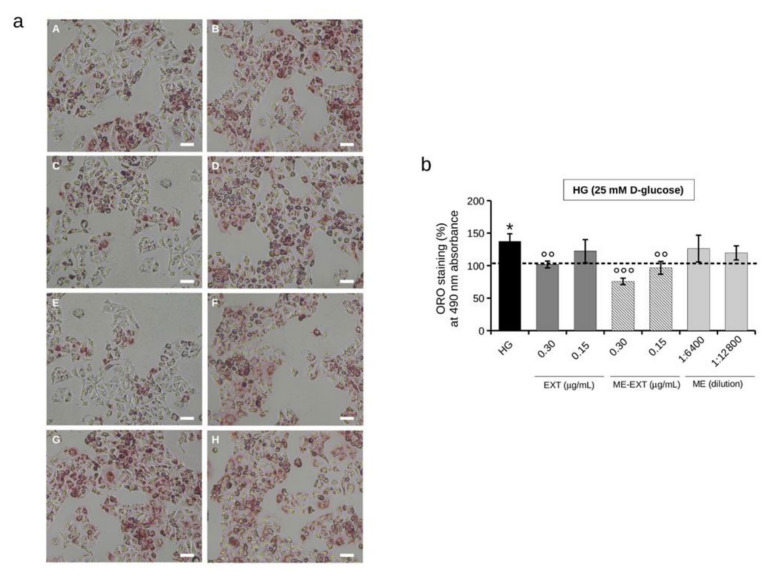
Intracellular lipid accumulation in HepG2 cells under normal glucose (NG, 5 mM) or high glucose (HG, 25 mM) conditions in the without or with EXT or ME-EXT (0.30 and 0.15 µg/mL) after 24 h treatment. (**a**) Representative image of ORO staining under the following conditions: (**A**) untreated cells exposed to NG conditions; (**B**) untreated cells exposed to HG conditions; (**C**) cells treated with EXT 0.30 µg/mL under HG conditions; (**D**) cells treated with EXT 0.15 µg/mL under HG conditions; (**E**) cells treated with ME-EXT (0.30 µg/mL of EXT loaded) under HG conditions; (**F**) cells treated with ME-EXT (0.15 µg/mL of EXT loaded) under HG conditions; (**G**) cells treated with ME vehicle 1:6400 under HG conditions; (**H**) cells treated with ME vehicle 1:12,800 under HG conditions. Scale bar = 100 µm. (**b**) Quantification of intracellular neutral lipid levels in cells treated under HG conditions with EXT (0.15 and 0.3 µg/mL), ME-EXT (0.15 and 0.3 µg/mL loaded EXT), and ME at the corresponding dilutions, by measuring ORO absorbance at 490 nm. Untreated cells exposed to HG were used as controls. Values are given in percentage terms compared to the same cell treatments performed under normal glucose conditions (NG, 5 mM D-glucose), represented on the graph by the dashed line. Data are reported as the mean ± SD of independent experiments. * *p* < 0.05 vs. NG untreated control cells; °° *p* < 0.01, °°° *p* < 0.001 vs. HG untreated control cells. Tukey’s HSD test.

The HG effect on intracellular lipid accumulation was evaluated by exposing HepG2 cells to HG for 24 h; the total neutral lipids were estimated using Oil Red O (ORO) staining (Figure 7A). As expected, HG conditions (25 mM D-glucose) significantly increased the neutral lipid accumulation by approximately 30% (130 ± 4%) in HepG2 cells compared to NG-exposed control cells (Figure 7B). The ability of EXT to prevent HG-induced lipid accumulation in HepG2 cells was then assessed (Figure 7A). As shown in Figure 7B, EXT 0.30 µg/mL inhibited HG-induced elevated intracellular neutral lipid levels by approximately 20% (112 ± 2%), whereas no effect was attributed to the lower dose of EXT 0.15 µg/mL. The literature attributes significant lipid-lowering effects in HepG2 cells to the many triterpenoids [56], proposing them as functional compounds to improve hepatic stress [57]. These results are, thus, in line with the scientific literature [56,57,58,59,60], and underline the potential of pentacyclic triterpene-rich EXT to lower HG-induced intracellular lipid levels.

TTPs benefits for human health are well known. Many factors, including absorption and metabolism, affect the in vivo efficacy in humans. There are a number of factors that can affect oral bioavailability, such as solubility and/or dissolution, permeation, first-pass metabolism, and pre-systemic excretion from the gut or liver [61]. Pentacyclic triterpenes are water-insoluble and lipophilic, which strongly influences their interactions with absorbent surface components.

Technology has enabled the development of new drug delivery systems that increase the bioavailability of herbal drugs [62]. Nanoformulations can improve the bioavailability and solubility of herbal formulations, protect them from physico-chemical degradation, enhance their therapeutic activity, improve their stability, and prolong administration [63]. In this study, the enhancing effect of the formulation (ME-EXT) on HG-induced intracellular lipid accumulation was demonstrated.

As shown in Figure 7A,B, cell treatment with ME-EXT significantly reduced, and in a dose-dependent manner, the HG-induced intracellular neutral lipid accumulation. That is, ME-EXT 0.30 µg/mL and 0.15 µg/mL reduced the accumulation of intracellular lipids in HepG2 cells in HG by approximately 50% (77 ± 4%) and 30% (100 ± 7%), respectively, compared to untreated HG cells. The ME vehicle, used as a control, had no effect on intracellular lipid accumulation.

The observed bio-enhancement of EXT could have been due to an increased internalization of EXT by the microemulsion. Recently, ME has been described as promoting the penetration of loaded compounds with poor solubility and permeability in some cell lines, due to the components of the formulation, particularly surfactants that act as enhancers [41,64].

### 2.7. The Effect of EXT and ME-EXT on High Glucose-Induced FASN Expression in HepG2 Cells

Cancer cells generate metabolic intermediates to meet rapid tumor growth demands, such as triglycerides and phospholipids, in response to an activated glycolytic flux [65].

**Figure 8:**
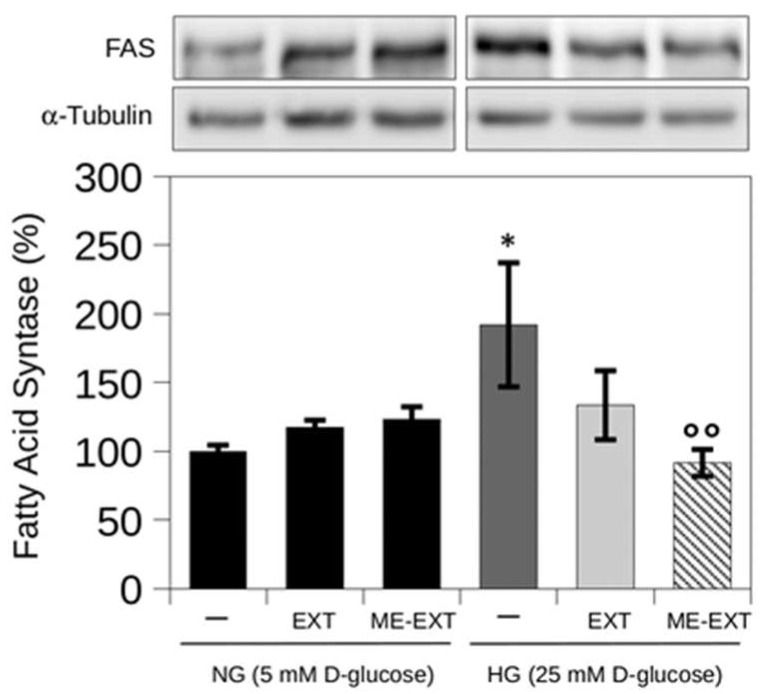
Representative image of FASN in HepG2 cells using a Western blot assay under NG (5 mM D-glucose) or HG (25 mM D-glucose) conditions, without or with EXT or ME-EXT (0.30 µg/mL). α-Tubulin (55 kDa) was used as a housekeeping protein in all expression analyses and as the loading control. A densitometric analysis was used to quantify chemiluminescence signals. Values are reported as the mean of independent experiments. Error bars represent standard errors. Statistical analysis was performed with a Kruskal–Wallis test: * *p* < 0.05 vs. untreated NG control cells (represented by “–“ in the black bar); °° *p* < 0.01 vs. untreated HG control cells (represented by “–“ in the dark grey bar).

Through glycolysis, glucose enters the cell and is transformed into pyruvate, then acetyl-CoA, which enters the Krebs cycle. Citrate from the Krebs cycle is exported to the cytoplasm in the presence of excess glucose, triggering the production of fatty acid synthesis intermediates. During this process, fatty acid synthase (FASN) catalyzes the de novo synthesis of fatty acids [66]. FASN plays a key role in the lipid synthesis in the liver by providing greater energy flexibility to meet energy needs [67,68]. A Western blot analysis of HepG2 cells exposed to HG for 24 h was used to monitor FASN expression levels (Figure 8). FASN expression levels increased by approximately 80% (183 ± 30%) during the 24-h exposure to HG, compared to NG conditions (Figure 8). In line with literature evidence, these results support an increase in hepatic FASN protein levels in response to glucose elevation [43,69]. Both mice with NAFLD induced by a high-fat diet and the liver of subjects with non-alcoholic fatty liver disease (NAFLD) showed an increased FASN expression [70]. Various malignant cells in vitro and in vivo have responded to pharmacological inhibition of FASN, but non-malignant cells have not responded. This represents a window for therapeutic intervention [70].

Therefore, here, the effect of EXT free (0.30 µg/mL) or ME-EXT (0.30 µg/mL) on FASN expression in HepG2 cells exposed to HG was evaluated. As shown in Figure 8, FASN levels were significantly reduced in EXT-treated HG cells by approximately 44% (139 ± 20%) at 24 h of treatment compared to untreated HG cells. Treatment with ME-EXT enhanced the effect of EXT on FASN expression. Indeed, the FASN levels were reduced by approximately 80% (94 ± 10%) in ME-EXT-treated HG cells compared to the untreated HG cells. These results agree with data obtained from the ORO assay on HepG2 and suggest that EXT reduced the lipid accumulation induced by high glucose levels through downregulation of HG-related de novo lipogenesis. This study also showed that the lipid-lowering effect of EXT under HG conditions in HepG2 cells can be enhanced through formulation. Microemulsions have been experimentally shown to facilitate the penetration of substances with poor solubility and bioavailability, due to the presence of tensides that increases permeability across the cell membrane [41,71]. In this study, ME was obtained as a homogeneous system, with high solubilizing power for TTPs and storage stability. This could realize a prolonged release of TTPs, compared with free EXT, with a significant increase in permeability, as evidenced by the PAMPA assay. Therefore, we can speculate that the improvement of EXT in preventing lipid accumulation when administered as ME may be due to increased internalization of EXT by the ME across cell membranes. Taken together, these results propose this ME-EXT formulation as a good vehicle for EXT delivery, due to its ability to overcome the drawbacks of lipophilic extracts.

## 3. Materials and Methods

### 3.1. Materials

Oleanolic acid (OA, >97%) and *Olea europaea* L. leaves extract (EXT) were supplied by Natac Biotech SL (Alcorcón, Madrid, Spain). Acetonitrile HPLC grade, Ethanol, Formic acid, Methanol HPLC grade, 1,7-octadiene, D-α-Tocopherol polyethylene glycol succinate (TPGS), cholesterol, and soy lecithin were purchased from Merck KgaA (Darmstadt, DA, Germany). Capryol 90 and Transcutol HP were supplied by Gattefossè sas (Saint-Priest, France). Water was from a Milli-Qplus system from Millipore (Milford, CT, USA). The dialysis kit was from Spectrum Laboratories, Inc. (Breda, The Netherlands). The PAMPA filter plate (pore size 0.45 μm) was purchased from Millipore Corporation (Tullagreen, Carrigtwohill, County Cork, Ireland). DMEM (Dulbecco’s Modified Eagle Medium), streptomycin, penicillin, L-glutamine, trypsin-EDTA solution, 1-(4,5-dimethylthiazol-2-yl)-3,5-diphenylformazan (MTT), and Oil Red O solution were purchased from Merck KgaA (Darmstadt, Germany). Cell culture plastics were provided by Sarstedt (Milan, Italy). Electrophoresis reagents were purchased from Bio-Rad (Bio-Rad Laboratories, Milano, Italy).

### 3.2. HPLC-DAD Analysis

A 1200 High-Performance Liquid Chromatograph equipped with a diode array detector (Agilent Technologies Italia Spa, Rome, Italy) was used for chromatographic determination. The analytical conditions have been previously reported [41]. The analytical column was a Luna Omega Polar C18 (150 × 4.6 mm, 3 μm, Agilent Technology, Santa Clara, CA, USA) coupled with a pre-column of the same phase, working at 25 °C. The eluent flow rate was 0.8 mL/min, with (A) acetonitrile and (B) water pH 3.2 (by formic acid) as mobile phase, and with an isocratic analytical method consisting of 85% A and 15% B for 25 min. The attribution of the peaks was performed at 210 nm using standards and referring to data previously published in the literature [13,72]. A standard OA methanol solution (2.0 mg/mL) and its dilutions were used to prepare the calibration curve. The coefficient of determination was 0.9999. The percentage of total TTPs was expressed as OA, using the following equation:

$$\mathrm{TTPs}\;\left(\%\right)=\frac{\left(A_{S}\times {\;C}_{\mathrm{std}}\right)}{\left(C_{\mathrm{sample}}\times {\;A}_{\mathrm{std}}\right)}\times {\;P}_{\mathrm{std}}$$

where A_s_ is the sum of the peak areas of the TTP in the EXT, C_std_ is the concentration (mg/mL) of the standard (OA), C_sample_ is the concentration (mg/mL) of the sample under analysis, A_std_ and P_std_ are the peak area and purity of the standard, respectively.

### 3.3. Pseudoternary Phase Diagram

The water titration method was used to construct the pseudoternary phase diagram. The surfactants were mixed at various ratios (Smix), and the pseudoternary phase diagrams were built using different weight oil-phase/Smix ratios. The lipophilic phase was maintained at 35 ± 2 °C under magnetic stirring and with water added while monitoring the change in sample appearance with each addition.

### 3.4. Preparation of Microemulsion (ME)

ME was prepared following the water titration method [73], by adding water dropwise to each oily phase/Smix blend. Transcutol HP and TPGS were selected as surfactants, with Capryol 90 as the oil. The lipophilic phase was obtained by mixing Transcutol HP and TPGS at a 9:1 ratio with Capryol 90, obtaining a final 9:1 Smix/Oil ratio. The components of the mixture were kept under magnetic stirring at 70 ± 2 °C, until complete dissolution of the TPGS. When the temperature reached 35 ± 2 °C, EXT (2 mg/mL) was added and the solution and the mixture were titrated with purified water. The resulting ME-EXT was maintained under magnetic stirring at 25 ± 2 °C for 10 min.

### 3.5. Particle Size and ζ-Potential Measurements

The particle size and ζ-potential of the ME-EXT were measured with light scattering techniques (DLS and ELS) using Ζetasizer Pro Red Label (Malvern Instruments, Malvern, UK). The hydrodynamic diameter and the particle size distribution (polydispersity index, PdI) were obtained using the ZS Xplorer software (Version 2.10) provided by Malvern. For each sample, values were obtained using the mean of three different measurements. The temperature was 25 °C.

### 3.6. Morphological Characterization

The samples were analyzed using a Gaia 3 Scanning Electron Microscope (Tescan s.r.o, Brno, Czech Republic). The FIB-SEM (focused ion beam-scanning electron micro-scope) electron beam used for TEM imaging had a voltage of 15 kV, operating in high-vacuum mode and with a bright-field TEM detector.

### 3.7. In Vitro Release Studies

An in vitro release study was carried out using the dialysis bag method (regenerated cellulose dialysis membranes, Spectrum Laboratories, Inc., Breda, The Netherlands, MWCO 12–14 kDa). The formulation was placed into a dialysis bag and immersed in 200 mL of EtOH:PBS (30:70 *v*/*v*) solution at 37 °C. The release of TTPs from ME-EXT was compared with that from the EXT solution, obtained by dissolving EXT in a 0.5% *w*/*v* solution of sodium dodecyl sulfate in water. Simulated gastric fluid (SGF) medium (pH 1.2, 2 h) and simulated intestinal fluid (SIF, pH 6.8, 6 h) were also used as release media [47,74]. At selected time points, an aliquot of each release medium was withdrawn and replaced with an equal volume of fresh solution. HPLC-DAD analysis was used to determine the TTP concentration. All experiments were repeated in triplicate.

### 3.8. Evaluation of Stability over Time

ME-EXT was stored at 4 °C and 25 °C for eight weeks. The changes in terms of particle size, homogeneity, ζ-potential, and TTPs concentration were assessed using DLS and HPLC-DAD analyses.

### 3.9. PAMPA Assay

The passive diffusion across an artificial membrane was checked using a 96-well MultiScreen IP filter plate for PAMPA (Millipore corporation). EtOH:PBS (30:70 *v*/*v*) solution (250 µL) was the acceptor medium. Each membrane was activated with a lecithin (10 g/L) and cholesterol (8 g/L) in 1,7-octadiene. After the deposition of this solution on the artificial membrane, 250 µL of EXT solution and appropriately diluted ME-EXT were added to each donor compartment. The plate was incubated at room temperature for 1 h. Then, the samples were withdrawn, diluted with methanol, centrifuged for 10 min at 14,000× *g*, and the TTP concentration was determined through HPLC-DAD analysis. The permeability was expressed as a coefficient of permeability Pe (cm/s) [41].

### 3.10. Cell Line and Culture Conditions

The human HepG2 hepatoma cell line was provided by the American Type Culture Collection (ATCC^®^). Cells were grown in a 5% CO_2_ humidified atmosphere at 37 °C in DMEM containing 10% FBS, 100 μg/mL streptomycin, 100 U/mL penicillin, and 2 mM L-glutamine (complete medium), and at a physiological glucose concentration (5 mM). HepG2 cells were appropriately propagated at 90% confluence using a 0.025% trypsin-0.5 mM EDTA solution and suitably diluted in growth medium. The here reported in vitro cell-based experiments were performed in complete medium containing 5 mM D-glucose (normal glucose, NG) or 25 mM D-glucose (high glucose, HG), in the presence of EXT or ME-EXT. Cells untreated or exposed to empty microemulsion (ME) were used as a control.

### 3.11. Cell Viability Assay

A 1-(4,5-dimethylthiazol-2-yl)-3,5-diphenylformazan (MTT) colorimetric assay was used to evaluate the HepG2 cell viability under different culture conditions. Briefly, HepG2 cells were seeded at a density of 3 × 10^4^ in 96-well plates in NG growth medium. Subsequently, the cells were treated under NG and HG conditions with EXT in the range of 0.04 to 0.30 µg/mL (*w*/*v*) or ME-EXT, at the corresponding concentrations of EXT. Cells untreated or treated with corresponding dilutions of the unformulated vehicle (ME) were used as controls. After 24 h of incubation, 100 µL of MTT solution (0.5 mg/mL) was added to each well and incubated for 1 h in the dark. Next, cells were lysed with dimethyl sulfoxide (100 μL/well), and the absorption values were measured using a microplate reader at 595 nm. The MTT assay was repeated in triplicate.

### 3.12. Intracellular Neutral Lipids Detection (Oil Red O Staining)

HepG2 cells were seeded at a density of 6 × 10^4^ in 24-well plates in NG growth medium and incubated overnight. Next, cells were treated in both NG and HG conditions with EXT in the range of 0.04 to 0.30 µg/mL (*w*/*v*) or ME-EXT, at the corresponding concentrations of EXT. Cells untreated or treated with corresponding dilutions of the empty ME were used as controls. After 24 h incubation, cells were fixed in 2% (*v*/*v*) paraformaldehyde for 10 min. After two PBS washes, wells were left to dry at 37 °C for a few minutes. At this point, a 60% (*v*/*v*) ORO solution in bi-distilled water was added at 200 µL/well and incubated at 37 °C, with stirring for 30 min. Subsequently, repeated washes in bi-distilled water were carried out to remove the excess dye. Images of intracellular lipid staining were captured using a Nikon TS-100 microscope equipped with a digital acquisition system (Nikon Digital Sight DS Fi-1; Nikon, Minato-ku, Tokyo, Japan). The staining intensity was measured by solubilizing the dye with isopropanol (200 µL/well). The absorption values were measured with an iMARK microplate reader (Bio-Rad Laboratories, Hercules, CA, USA) at a wavelength of 490 nm [75]. Cell viability values were used to normalize the ORO assay data. Values are reported in terms of the percentage with respect to untreated control cells.

### 3.13. Fatty Acid Synthase (FASN) Detection Using Western Blot Assay

HepG2 cells (15 × 10^4^ cells/well) were cultured in 6-well plates for 24 h. The cells were then exposed to NG and HG conditions in the absence or presence of EXT at a concentration of 0.30 µg/mL (*w*/*v*) or ME-EXT, at the corresponding concentration of EXT for 24 h. A Laemmli buffer solution containing Tris-HCl (62.5 mM, pH 6.8), 10% (*w*/*v*) SDS, and 25% (*w*/*v*) glycerol was used to lyse the cells. Lysates were centrifuged at 4 °C for 1 min at 12,000× *g*. The total protein concentration of each sample was determined using a BCA protein assay. Then, 30 μg protein from each sample was mixed with 5% (*v*/*v*) β-mercaptoethanol and bromophenol blue and heated at 95 °C for 5 min. Protein samples were electrophoretically separated on 12% SDS-polyacrylamide gels and blotted onto PVDF membranes (0.45 μm). After a saturation step with a BSA blocking buffer (5% (*w*/*v*) BSA in 0.1% (*v*/*v*) PBS-Tween^®^-20), the membranes were incubated overnight at 4 °C with FASN primary antibody (Rabbit IgG, Cell Signaling) diluted 1:1000, and α-Tubulin (Rabbit IgG, Genetex) diluted 1:1000 in the blocking buffer. After three washes in 0.1% (*v*/*v*) PBS-Tween^®^-20 solution, HRP-linked secondary antibodies of goat anti-rabbit IgG (1:10,000) (Invitrogen, Waltham, MA, USA) were added to each membrane for 1 h at room temperature. After three washes in 0.5% (*v*/*v*) PBS-Tween^®^-20, Clarity Western ECL solution was used to detect protein bands, using an Amersham^TM^ 600 Imager imaging system (GE Healthcare Life Science, Pittsburgh, PA, USA). Quantity One (version 4.6.6, Bio-Rad) was used as the instrument for densitometric analysis of the protein bands.

## 4. Conclusions

In this study, a microemulsion was proposed to improve the oral administration of a TTP-enriched extract obtained from olive leaves. The ME was obtained as homogeneous dispersed-phase droplets with sizes within the appropriate range for oral application, with a significant increase in the aqueous solubility of EXT. Physical and chemical analyses of ME over two months at two different temperatures showed the high stability of the formulation. The ME allowed a prolonged release of EXT, and PAMPA permeation studies revealed that the formulation significantly increased their permeability. In addition, cell-based experiments showed that the EXT was able to reduce lipid accumulation in HepG2 cells by acting on de novo lipogenesis. Interestingly, the ME-EXT formulation was shown to potentiate the inhibitory activity of EXT. The effect of the TTP-enriched extract on the reduction in lipid accumulation and the bio-enhancement achieved by the ME formulation were evaluated on a human HepG2 hepatocarcinoma cell line, a good model of liver cancer. In light of these findings, this work underscores the potential of ME as a promising and effective oral formulation for the delivery of lipophilic extracts. However, it will be necessary to test the oral application of ME-EXT through in vivo animal models to assess the influence of the formulation on pharmacokinetics parameters and its potential clinical value for the treatment of chronic and noncommunicable diseases.

## Data Availability

The data presented in this study are available on request from the corresponding author.

## Supplementary Materials

The supporting information can be downloaded at https://www.mdpi.com/article/10.3390/ijms241512113/s1.

## References

[1] Markhali F.S., Teixeira J.A., Rocha C.M.R. (2020). Olive Tree Leaves—A Source of Valuable Active Compounds. Processes.

[2] Agatonovic-Kustrin S., Gegechkori V., Mohammed E.U.R., Ku H., Morton D.W. (2022). Isolation of Bioactive Pentacyclic Triterpenoid Acids from Olive Tree Leaves with Flash Chromatography. Appl. Sci..

[3] Stiti N., Hartmann M.A. (2012). Nonsterol Triterpenoids as Major Constituents of *Olea europaea*. J. Lipids.

[4] El S.N., Karakaya S. (2009). Olive tree (*Olea europaea*) leaves: Potential beneficial effects on human health. Nutr. Rev..

[5] Rufino-Palomares E.E., Pérez-Jiménez A., García-Salguero L., Mokhtari K., Reyes-Zurita F.J., Peragón-Sánchez J., Lupiáñez J.A. (2022). Nutraceutical Role of Polyphenols and Triterpenes Present in the Extracts of Fruits and Leaves of *Olea europaea* as Antioxidants, Anti-Infectives and Anticancer Agents on Healthy Growth. Molecules.

[6] Rufino-Palomares E.E., Perez-Jimenez A., Reyes-Zurita F.J., Salguero L.G., Mokhtari K., Herrera-Merchan A., Medina P.P., Peragon J., Lupianez J.A. (2015). Anti-cancer and Anti-angiogenic Properties of Various Natural Pentacyclic Tri-terpenoids and Some of their Chemical Derivatives. Curr. Org. Chem..

[7] Guinda Á., Rada M., Delgado T., Gutiérrez-Adánez P., Castellano J.M. (2010). Pentacyclic Triterpenoids from Olive Fruit and Leaf. J. Agric. Food Chem..

[8] Jeong D.W., Kim Y.H., Kim H.H., Ji H.Y., Yoo S.D., Choi W.R., Lee S.M., Han C.K., Lee H.S. (2007). Dose-linear pharmacokinetics of oleanolic acid after intravenous and oral administration in rats. Biopharm. Drug Dispos..

[9] Song M., Hang T.J., Wang Y., Jiang L., Wu X.L., Zhang Z., Shen J., Zhang Y. (2006). Determination of oleanolic acid in human plasma and study of its pharmacokinetics in Chinese healthy male volunteers by HPLC tandem mass spectrometry. J. Pharm. Biomed. Anal..

[10] De la Torre R., Carbó M., Pujadas M., Biel S., Mesa M.D., Covas M.I., Expósito M., Espejo J.A., Sanchez-Rodriguez E., Díaz-Pellicer P. (2020). Pharmacokinetics of maslinic and oleanolic acids from olive oil—Effects on endothelial function in healthy adults. A randomized, controlled, dose-response study. Food Chem..

[11] Sánchez-González M., Colom H., Lozano-Mena G., Juan M.E., Planas J.M. (2014). Population pharmacokinetics of maslinic acid, a triterpene from olives, after intravenous and oral administration in rats. Mol. Nutr. Food Res..

[12] Jinhua W. (2019). Ursolic acid: Pharmacokinetics process in vitro and in vivo, a mini review. Arch. Pharm..

[13] Rada M., Ruiz-Gutiérrez V., Guinda Á. (2011). Determination of triterpenic acids in human serum by high-performance liquid chromatography: Triterpenoid interaction with serum protein. J. Agric. Food Chem..

[14] Sánchez-Quesada C., López-Biedma A., Warleta F., Campos M., Beltrán G., Gaforio J.J. (2013). Bioactive properties of the main triterpenes found in olives, virgin olive oil, and leaves of *Olea europaea*. J. Agric. Food Chem..

[15] Hwang Y.J., Song J., Kim H.R., Hwang K.A. (2014). Oleanolic acid regulates NF-κB signaling by suppressing MafK expression in RAW 264.7 cells. BMB Rep..

[16] Tsai S.J., Yin M.C. (2008). Antioxidative and anti-inflammatory protection of oleanolic acid and ursolic acid in PC12 cells. J. Food Sci..

[17] Castellano J.M., Ramos-Romero S., Perona J.S. (2022). Oleanolic Acid: Extraction, Characterization and Biological Activity. Nutrients.

[18] Castellano J.M., Guinda A., Delgado T., Rada M., Cayuela J.A. (2013). Biochemical basis of the antidiabetic activity of oleanolic acid and related pentacyclic triterpenes. Diabetes.

[19] Bu Y., Shi T., Meng M., Kong G., Tian Y., Chen Q., Yao X., Feng G., Chen H., Lu Z. (2011). A novel screening model for the molecular drug for diabetes and obesity based on tyrosine phosphatase Shp2. Bioorg. Med. Chem. Lett..

[20] Buus N.H., Hansson N.C., Rodriguez-Rodriguez R., Stankevicius E., Andersen M.R., Simonsen U. (2011). Antiatherogenic effects of oleanolic acid in apolipoprotein E knockout mice. Eur. J. Pharmacol..

[21] Erdmann J., Kujaciński M., Wiciński M. (2021). Beneficial Effects of Ursolic Acid and Its Derivatives-Focus on Potential Biochemical Mechanisms in Cardiovascular Conditions. Nutrients.

[22] Kang Y.M., Kim H.M., Lee M., An H.J. (2021). Oleanolic Acid Alleviates Atopic Dermatitis-like Responses In Vivo and In Vitro. Int. J. Mol. Sci..

[23] Liu L., Wang X. (2007). Improved dissolution of oleanolic acid with ternary solid dispersions. AAPS PharmSciTech.

[24] Yang J., Li X., Yang H., Long C. (2021). Oleanolic Acid Improves the Symptom of Renal Ischemia Reperfusion Injury via the PI3K/AKT Pathway. Urol. Int..

[25] Alhadrami H.A., Sayed A.M., Sharif A.M., Azhar E.I., Rateb M.E. (2021). Olive-Derived Triterpenes Suppress SARS CoV-2 Main Protease: A Promising Scaffold for Future Therapeutics. Molecules.

[26] Ghante M.H., Jamkhande P.G. (2019). Role of Pentacyclic Triterpenoids in Chemoprevention and Anticancer Treatment: An Overview on Targets and Underling Mechanisms. J. Pharmacopunct..

[27] Furtado N.A.J.C., Pirson L., Edelberg H., Miranda L.M., Loira-Pastoriza C., Preat V., Larondelle Y., André C.M. (2017). Pentacyclic Triterpene Bioavailability: An Overview of In Vitro and In Vivo Studies. Molecules.

[28] Banerjee S., Bose S., Mandal S.C., Dawn S., Sahoo U., Ramadan M.A., Mandal S.K. (2019). Pharmacological Property of Pentacyclic Triterpenoids. Egypt. J. Chem..

[29] Wang X., Bai H., Zhang X., Liu J., Cao P., Liao N., Zhang W., Wang Z., Hai C. (2013). Inhibitory effect of oleanolic acid on hepatocellular carcinoma via ERK-p53-mediated cell cycle arrest and mitochondrial-dependent apoptosis. Carcinogenesis.

[30] Jannus F., Medina-O’Donnell M., Rivas F., Díaz-Ruiz L., Rufino-Palomares E.E., Lupiáñez J.A., Parra A., Reyes-Zurita F.J. (2020). A Diamine-PEGylated Oleanolic Acid Derivative Induced Efficient Apoptosis through a Death Receptor and Mitochondrial Apoptotic Pathway in HepG2 Human Hepatoma Cells. Biomolecules.

[31] Llovet J.M., Kelley R.K., Villanueva A., Singal A.G., Pikarsky E., Roayaie S., Lencioni R., Koike K., Zucman-Rossi J., Finn R.S. (2021). Hepatocellular carcinoma. Nat. Rev. Dis. Primers.

[32] Forner A., Reig M., Bruix J. (2018). Hepatocellular carcinoma. Lancet.

[33] Cotter T.G., Rinella M. (2020). Nonalcoholic Fatty Liver Disease 2020: The State of the Disease. Gastroenterology.

[34] Antwi S.O., Craver E.C., Nartey Y.A., Sartorius K., Patel T. (2022). Metabolic Risk Factors for Hepatocellular Carcinoma in Patients with Nonalcoholic Fatty Liver Disease: A Prospective Study. Cancers.

[35] Paul B., Lewinska M., Andersen J.B. (2022). Lipid alterations in chronic liver disease and liver cancer. JHEP Rep..

[36] Currie E., Schulze A., Zechner R., Walther T.C., Farese R.V. (2013). Cellular fatty acid metabolism and cancer. Cell Metab..

[37] Hao Q., Li T., Zhang X., Gao P., Qiao P., Li S., Geng Z. (2014). Expression and roles of fatty acid synthase in hepatocellular carcinoma. Oncol. Rep..

[38] Li L., Che L., Tharp K.M., Park H.M., Pilo M.G., Cao D., Cigliano A., Latte G., Xu Z., Ribback S. (2016). Differential requirement for de novo lipogenesis in cholangiocarcinoma and hepatocellular carcinoma of mice and humans. Hepatology.

[39] Reveron-Thornton R.F., Teng M.L.P., Lee E.Y., Tran A., Vajanaphanich S., Tan E.X., Nerurkar S.N., Ng R.X., The R., Tripathy D.P. (2022). Global and regional long-term survival following resection for HCC in the recent decade: A meta-analysis of 110 studies. Hepatol. Commun..

[40] Piazzini V., Monteforte E., Luceri C., Bigagli E., Bilia A.R., Bergonzi M.C. (2017). Nanoemulsion for improving solubility and permeability of Vitex agnus-castus extract: Formulation and in vitro evaluation using PAMPA and Caco-2 approaches. Drug Deliv..

[41] De Stefani C., Vasarri M., Salvatici M.C., Grifoni L., Quintela J.C., Bilia A.R., Degl’Innocenti D., Bergonzi M.C. (2022). Microemulsions Enhance the In Vitro Antioxidant Activity of Oleanolic Acid in RAW 264.7 Cells. Pharmaceutics.

[42] Lodovichi J., Landucci E., Pitto L., Gisone I., D’Ambrosio M., Luceri C., Salvatici M.C., Bergonzi M.C. (2022). Evaluation of the increase of the thymoquinone permeability formulated in polymeric micelles: In vitro test and in vivo toxicity assessment in Zebrafish embryos. Eur. J. Pharm. Sci..

[43] Vasarri M., Barletta E., Stio M., Bergonzi M.C., Galli A., Degl’Innocenti D. (2023). Ameliorative Effect of *Posidonia oceanica* on High Glucose-Related Stress in Human Hepatoma HepG2 Cells. Int. J. Mol. Sci..

[44] Williamson E.M. (2001). Synergy and other interactions in phytomedicins. Phytomedicins.

[45] Lee O.H., Lee B.Y. (2010). Antioxidant and antimicrobial activities of individual and combined phenolics in *Olea europaea* leaf extract. Bioresour. Technol..

[46] Porter C.J.H., Trevaskis N.L., Charman W.N. (2007). Lipids and lipid-based formulations: Optimizing the oral delivery of lipophilic drugs. Nat. Rev. Drug Discov..

[47] Cecchi L., Piazzini V., D’Ambrosio M., Luceri C., Rocco F., Innocenti M., Vanti G., Mulinacci N., Bergonzi M.C. (2020). Formulation of a Phenol-Rich Extract from Unripe Olives (*Olea europaea* L.) in Microemulsion to Improve Its Solubility and Intestinal Permeability. Molecules.

[48] Porter C.J., Pouton C.W., Cuine J.F., Charman W.N. (2008). Enhancing intestinal drug solubilisation using lipid-based delivery systems. Adv. Drug Deliv. Rev..

[49] Yin Y.M., Cui F.D., Mu C.F., Choi M.K., Kim J.S., Chung S.J., Shim C.K., Kim D.D. (2009). Docetaxel microemulsion for enhanced oral bioavailability: Preparation and in vitro and in vivo evaluation. J. Control Release.

[50] Piazzini V., Rosseti C., Bigagli E., Luceri C., Bilia A.R., Bergonzi M.C. (2017). Prediction of Permeation and Cellular Transport of Silybum marianum Extract Formulated in a Nanoemulsion by Using PAMPA and Caco-2 Cell Models. Planta Med..

[51] Lin Y.L., Shen Q., Katsumi H., Okada N., Fujita T., Jiang X.H., Yamamoto A. (2007). Effects of Labrasol and other pharmaceutical excipients on the intestinal transport and absorption of rhodamine123, a P-glycoprotein substrate, in rats. Biol. Pharm. Bull..

[52] Campos C. (2012). Chronic hyperglycemia and glucose toxicity: Pathology and clinical sequelae. Postgrad. Med..

[53] Cheng H., Wang M., Su J., Li Y., Long J., Chu J., Wan X., Cao Y., Li Q. (2022). Lipid Metabolism and Cancer. Life.

[54] Khan W., Augustine D., Rao R.S., Patil S., Awan K.H., Sowmya S.V., Haragannavar V.C., Prasad K. (2021). Lipid metabolism in cancer: A systematic review. J. Carcinog..

[55] Zhang Y., Takemori H., Wang C., Fu J., Xu M., Xiong L., Li N., Wen X. (2017). Role of salt inducible kinase 1 in high glucose-induced lipid accumulation in HepG2 cells and metformin intervention. Life Sci..

[56] Yang W., Zheng X., Bai J., Zhong P., Tan S., Zeng W., Chen J., Sun Z., Liu Z., Jin J. (2023). Triterpenoids from the genus Ilex attenuate free fatty acid-induced lipid accumulation in HepG2 cells by regulating lipid metabolism disorder and the AMPK signalling pathway. J. Ethnopharmacol..

[57] Yang H.M., Yin Z.Q., Zhao M.G., Jiang C.H., Zhang J., Pan K. (2018). Pentacyclic triterpenoids from *Cyclocarya paliurus* and their antioxidant activities in FFA-induced HepG2 steatosis cells. Phytochemistry.

[58] Sharma H., Kumar P., Deshmukh R.R., Bishayee A., Kumar S. (2018). Pentacyclic triterpenes: New tools to fight metabolic syndrome. Phytomedicine.

[59] Balcazar N., Betancur L.I., Muñoz D.L., Cabrera F.J., Castaño A., Echeverri L.F., Acin S. (2021). Ursolic Acid Lactone Obtained from *Eucalyptus tereticornis* Increases Glucose Uptake and Reduces Inflammatory Activity and Intracellular Neutral Fat: An In Vitro Study. Molecules.

[60] Liu G., Cui Z., Gao X., Liu H., Wang L., Gong J., Wang A., Zhang J., Ma Q., Huang Y. (2021). Corosolic acid ameliorates non-alcoholic steatohepatitis induced by high-fat diet and carbon tetrachloride by regulating TGF-β1/Smad2, NF-κB, and AMPK signaling pathways. Phytother. Res..

[61] Azman M., Sabri A.H., Anjani Q.K., Mustaffa M.F., Hamid K.A. (2022). Intestinal Absorption Study: Challenges and Absorption Enhancement Strategies in Improving Oral Drug Delivery. Pharmaceuticals.

[62] Bonifácio B.V., Silva P.B., Ramos M.A., Negri K.M., Bauab T.M., Chorilli M. (2014). Nanotechnology-based drug delivery systems and herbal medicines: A review. Int. J. Nanomed..

[63] Dewi M.K., Chaerunisaa A.Y., Muhaimin M., Joni I.M. (2022). Improved Activity of Herbal Medicines through Nanotechnology. Nanomaterials.

[64] Constantinides P.P. (1995). Lipid microemulsions for improving drug dissolution and oral absorption: Physical and biopharmaceutical aspects. Pharm. Res..

[65] Vaupel P., Schmidberger H., Mayer A. (2019). The Warburg effect: Essential part of metabolic reprogramming and central contributor to cancer progression. Int. J. Radiat. Biol..

[66] Solinas G., Borén J., Dulloo A.G. (2015). De novo lipogenesis in metabolic homeostasis: More friend than foe?. Mol. Metab..

[67] Jensen-Urstad A.P., Semenkovich C.F. (2012). Fatty acid synthase and liver triglyceride metabolism: Housekeeper or messenger?. Biochim. Biophys. Acta.

[68] Pouton C.W. (2006). Formulation of poorly water-soluble drugs for oral administration: Physicochemical and physiological issues and the lipid formulation classification system. Eur. J. Pharm. Sci..

[69] Villanueva-Ortega E., Méndez-García L.A., Garibay-Nieto G.N., Laresgoiti-Servitje E., Medina-Bravo P., Olivos-García A., Muñoz-Ortega M.H., Ventura-Juárez J., Escobedo G. (2020). Growth hormone ameliorates high glucose-induced steatosis on in vitro cultured human HepG2 hepatocytes by inhibiting de novo lipogenesis via ChREBP and FAS suppression. Growth Horm. IGF Res..

[70] Fhu C.W., Ali A. (2020). Fatty Acid Synthase: An Emerging Target in Cancer. Molecules.

[71] Zheng Y., Xu G., Ni Q., Wang Y., Gao Q., Zhang Y. (2022). Microemulsion Delivery System Improves Cellular Uptake of Genipin and Its Protective Effect against Aβ1-42-Induced PC12 Cell Cytotoxicity. Pharmaceutics.

[72] Guinda A., Castellano J.M., Santos-Lozano J.M., Delgado-Hervás T., Gutiérrez-Adánez P., Rada M. (2015). Determination of major bioactive compounds from olive leaf. LWT-Food Sci. Technol..

[73] Ghosh P.K., Murthy R.S. (2006). Microemulsions: A potential drug delivery system. Curr. Drug Deliv..

[74] USP 25 the United States Pharmacopeia (2002). NF 20 The National Formulary.

[75] Vasarri M., Barletta E., Degl’Innocenti D. (2021). *Posidonia oceanica* (L.) Delile Extract Reduces Lipid Accumulation through Autophagy Activation in HepG2 Cells. Pharmaceuticals.

