# Supplementary material for: Pentacyclic Triterpenes from Olive Leaves Formulated in Microemulsion: Characterization and Role in De Novo Lipogenesis in HepG2 Cells
Source: Int J Mol Sci. 2023 Jul 28;24(15):12113. doi: 10.3390/ijms241512113 (PMC10419275; doi:10.3390/ijms241512113)

Supplementary Materials

Figure S1. Pseudo-ternary phase diagram of ME-EXT.

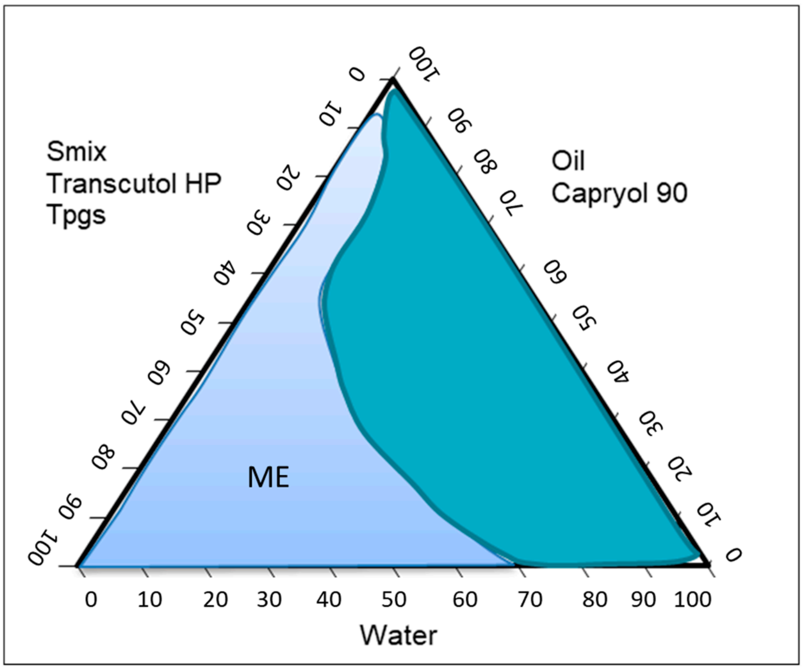

Figure S2. TEM analysis of ME-EXT. Bar: 200 nm.

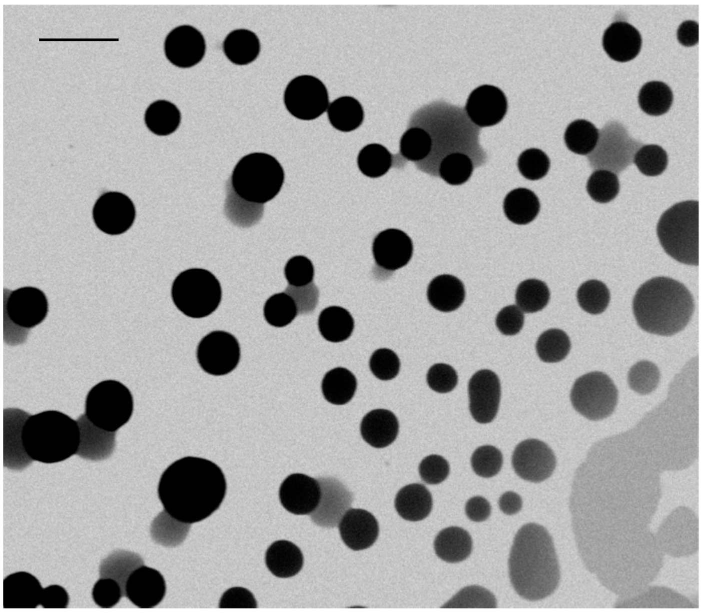

Figure S3. In vitro release profile of TTPs from the ME-EXT in SGF (a, pH 1.2, 2h) and (SIF) (b, pH 6.8, 6 h). Each value is the mean ± SD of three separate determinations.

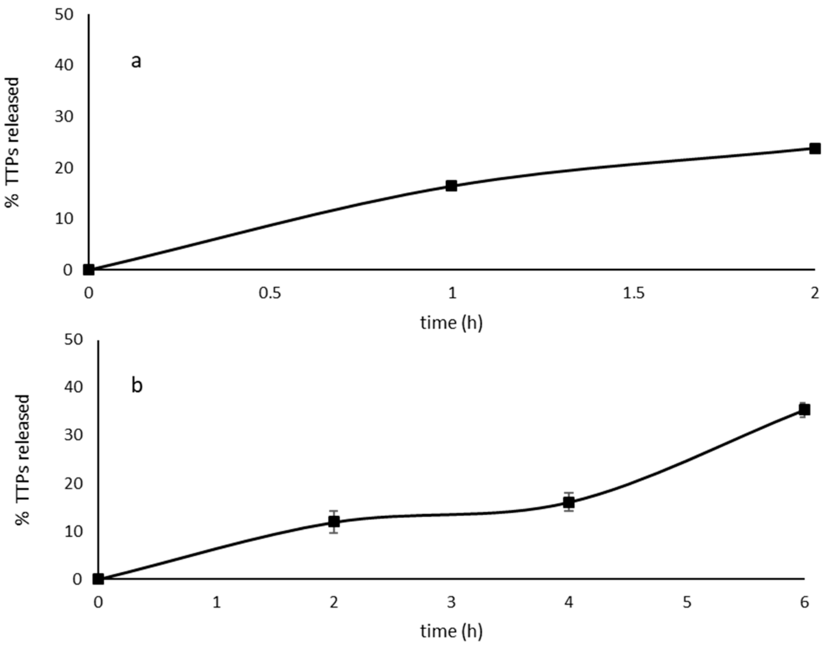

Figure S4. Cell viability of HepG2 human hepatoma cells treated empty ME at various dilutions, under high-glucose conditions (HG, 25 mM D-glucose) for 24h. Untreated cells exposed to HG are used as control. The values are given as percentages compared to the same cell treatments carried out under normal glucose conditions normal (NG, 5 mM D-glucose) represented on the graph by the dotted line. The data are obtained from the mean ± standard deviation of three experiments

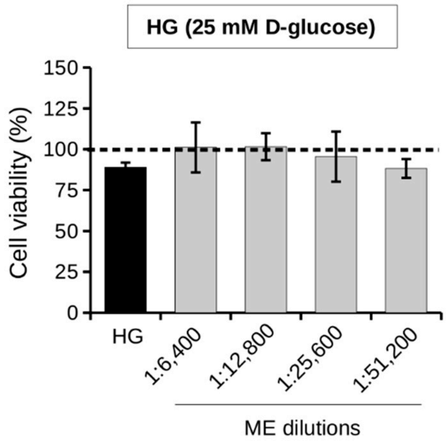

Supplement: Supplementary file 1 [file ijms-24-12113-s001.zip › ijms-2476479-supplementary.pdf]
